# Providencia rettgeri Infection Compromising Post-Burn Recovery: A Lesson in the Importance of Follow-Up Care

**DOI:** 10.7759/cureus.25450

**Published:** 2022-05-29

**Authors:** Mallorie L Huff, Sigrid Blome-Eberwein

**Affiliations:** 1 Department of Surgery, Division of Burn Surgery, University of South Florida Morsani College of Medicine/Lehigh Valley Health Network Campus, Allentown, USA

**Keywords:** patient follow-up, social determinants of health (sdoh), split thickness skin graft, gram negative infection, burn grafting, burn wound care, providencia rettgeri

## Abstract

Early burn excision and skin grafting have been essential to improving patient outcomes following a burn injury. However, there remain significant barriers to recovery for burn patients, especially in the post-discharge period, as follow-up care is essential to the timely identification of complications. While the infection is a common complication of a post-burn wound, *Providencia rettgeri* is an uncommon bacterial pathogen that rarely causes wound infections. Although *P. rettgeri *has been infrequently reported as a cause of wound infections, it is a pathogen with growing clinical significance due to innate and acquired mechanisms of antimicrobial resistance that may complicate patient treatment. While our report describes the clinical outcome of a patient with a wound infection with *Providencia rettgeri*, it also represents a case that underscores the importance of social determinants of health in the care of burn patients. This is a case report of an elderly male who sustained burns to his bilateral arms and back and was subsequently readmitted to our burn unit for graft loss associated with a *Providencia rettgeri* wound infection. His readmission required multiple operations to resect necrotic tissue and nonviable graft due to delayed wound healing and incomplete graft take. Inadequate access to transportation led to significantly delayed follow-up for this patient.

## Introduction

*Providencia rettgeri*, formerly *Proteus rettgeri*, is an uncommonly encountered, urease-producing gram-negative bacillus. In the environment, *Providencia *species exist as ubiquitous organisms in the soil and as colonizing flora of certain vertebrate and invertebrate species [1,2]. There are five species within the Providencia genus: *Providencia stuartii*, *Providencia rettgeri*, *Providencia alcalifaciens*, *Providencia heimbachae*, and Providencia rustigianii. Within the genus, *P. stuartii*, *P. rettgeri*, and *P. alacalifaciens* cause clinical disease [3]. In a clinical setting, *P. rettgeri* is typically seen as a causative agent of urinary tract infections in hospitalized patients, famously producing “purple bag syndrome” in elderly patients with indwelling urinary catheters due to innate indoxyl sulfatase activity [1,4]. However, *P. rettgeri* has also been uncommonly identified in peritoneal fluid [5,6], wounds [7,8], and as a causative agent of neonatal sepsis [9]. Of the Providencia species, *P. stuartii* has been mostly described as a complicating factor in burn wounds, with *P. stuartii* colonization portending significant morbidity in burn patients with increased length of stay and amount of debridement required [10].

In a burn patient population, timely follow-up after discharge is essential. Following discharge, this population requires significant physical and psychological support to recover from a burn injury. While early burn wound excision with subsequent split-thickness skin grafts (STSG) have substantially improved patient outcomes and reduced mortality, burn patients still face significant challenges in the immediate period following discharge [11]. As delayed wound healing, burn wound infection, pain, and hypertrophic scarring are commonly encountered after burn trauma, follow-up appointments are essential for identifying problems and intervening early [11]. Continuous communication and follow-up care through patient telecommunication or regular appointments have been shown to improve patient-reported quality of life as well as self-reported physical and psychological health [12,13]. In this case report, we describe a case of polymicrobial wound infection with *Providencia rettgeri* in a burn patient that led to significant morbidity and was complicated by inadequate access to transportation.

## Case presentation

**Figure 1:**
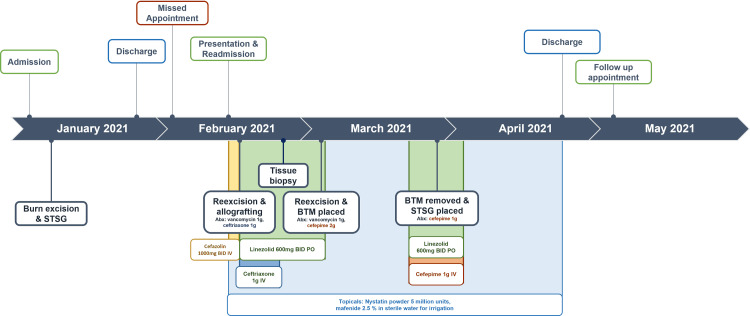
Timeline of patient care episodes, indicating admission, discharge, appointments, antibiotics given, and surgical interventions. STSG: split thickness skin graft, BTM: biodegradable temporizing matrix

A 72-year-old man presented with 7% total body surface area (TBSA) mixed partial and full-thickness depth burns to his back and bilateral upper extremities, reportedly obtained when he awoke next to a space heater early in the morning. He was initially transferred from an outside institution to our burn center for definitive burn care and had a significant past medical history of chronic kidney disease, coronary artery disease, congestive heart failure, diabetes mellitus, and gout. On day four of this admission, he underwent excision of his bilateral upper extremity burns with allograft application, followed by meshed STSG placement six days later. Allograft was used as a temporizing coverage to promote wound re-epithelization and improve the wound bed for later graft take. His back was managed conservatively with wound care and dressing changes. He had substantial postoperative pain, requiring a multimodal regimen to obtain adequate control. After a 25-day inpatient stay, he was discharged home with wound care. Extensive education was given, describing signs and symptoms of infection, burn care, and pain medication regimen. A follow-up appointment was scheduled for one week after discharge, and the importance of follow-up with the Burn Recovery Center was emphasized (Figure 1).

One week following hospital discharge, the patient did not come to his appointment due to difficulty obtaining transportation. His insurance provider had a 60-mile limitation for transportation services, and he lacked access to transportation or social support at home to bring him to his appointment. He lived 84 miles from our facility without any closer burn centers available for care. Transportation to the burn center was coordinated as a combined effort between the patient’s primary care office, our burn center, case management, and the insurance provider, with the patient finally being evaluated in person at our center 19 days after discharge.

**Figure 2:**
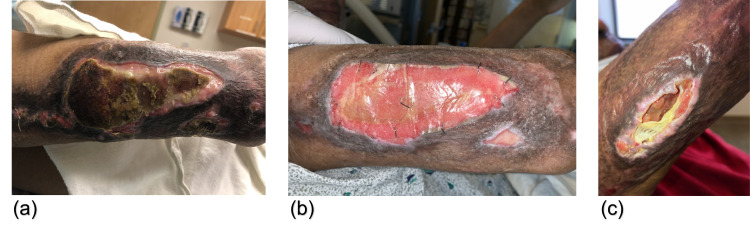
Appearance of right elbow wound (A) on readmission, (B) mid-course with BTM in place, and (C) post-discharge on outpatient follow-up. BTM: biodegradable temporizing matrix

**Figure 3:**
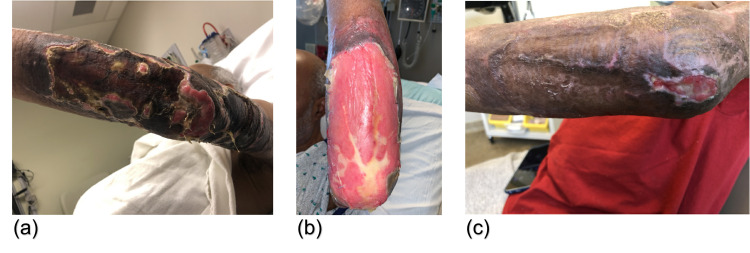
Appearance of left elbow wound (A) on readmission, (B) mid-course with BTM in place, and (C) post-discharge on outpatient follow-up. BTM: biodegradable temporizing matrix

On presentation, he exhibited substantial graft loss with uncontrolled pain in his arm wounds. On the right elbow, there was 50% graft loss with clear dermal step-offs to granulation tissue alongside centralized, adherent desiccated necrotic tissue with small areas of purulence (Figure 2). The left elbow exhibited 100% graft loss with adherent, desiccated necrotic tissue with purulence and large areas of hypergranulated tissue (Figure 3). Neither site demonstrated erythema or warmth. His back showed epithelialized wound beds with soft, flat underlying post-injury hyperemia. The bilateral anterior thigh donor sites were primarily healed with superficial open wounds from sheering. He was admitted to our inpatient burn unit for wound care, re-grafting of his bilateral arms, and pain control. Wound cultures were taken, a preliminary nasal screen for all *staphylococcus *species returned positive, and he was begun on intravenous cefazolin (Figure 1). Throughout his longitudinal course of care, topical treatments were maintained, including nystatin powder, mafenide irrigation, mupirocin ointment, and bacitracin zinc-polymyxin B ointment (Figure 1).

On day four of readmission, he underwent re-excision of the nonviable tissue on the upper extremities (Figure 1). Per institutional burn center protocol, he received cefepime perioperatively as empiric methicillin-resistant staphylococcus aureus (MRSA) and *Pseudomonas *coverage (Figure 1). There was deep tissue involvement to the depth of the triceps on both bilateral upper extremities and into the volar compartment on the left lower arm. Given his presentation with overtly necrotic graft and concern for wound infection, allograft was utilized as a temporizing coverage over repeat STSG. Wound cultures returned, showing polymicrobial infection with MRSA, methicillin-sensitive staphylococcus aureus (MSSA), and *Providencia rettgeri* (Table 1). A 14-day course of oral linezolid was initiated to cover MRSA, and IV ceftriaxone was initiated to cover *P. rettgeri* (Figure 1). Trimethoprim-sulfamethoxazole is typically not used as a first-line medication at our burn center out of precaution for Stevens-Johnson syndrome/toxic epidermal necrolysis. A week following the procedure, there was minimal graft adherence. His wound appearance, in the context of overt graft necrosis on presentation and failed graft, taken despite antibiotic coverage, prompted concern for pyoderma gangrenosum or other underlying rheumatologic diseases. Punch biopsy was performed, showing nonspecific superficial mild neutrophilic and lymphocytoplasmic inflammation, most consistent with ecthyma. An extensive workup was similarly negative for any acute rheumatologic process.

**Table 1:** Antibiogram for species identified in wound culture indicating sensitivity, resistance, and MIC MIC: minimum inhibitory concentration

	Providencia rettgeri		Staphylococcus aureus		*Methicillin-resistant* *Staphylococcus aureus*	
Ampicillin		R				
Ampicillin/Sulbactam	16	I				
Aztreonam	<=1	S				
Cefazolin	>=64	R				
Cefepime	<=1	S				
Ceftazidime	<=1	S				
Ceftriaxone	<=1	S				
Ciprofloxacin	<=0.25	S				
Clindamycin			0.25	S	>=4	R
Daptomycin			0.25	S	0.25	S
Erythromycin			<=0.25	S	>=8	R
Gentamicin	<=1	S				
Linezolid			2	S	2	S
Meropenem	<=0.25	S				
Oxacillin (Nafcillin)			0.5	S	>=4	R
Penicillin			>=0.5	R		
Piperacillin/Tazobactam	<=4	S				
Rifampin			<=0.5	S	<=0.5	S
Tetracycline			<=1	S	<=1	S
Trimethoprim/Sulfamethoxazole	<=20	S	<=10	S	<=10	S
Vancomycin			<=0.5	S	<=0.5	S
S = Susceptible; I = Intermediate; R= Resistant						

He continued to have difficulties with wound healing and returned to the operating room for re-excision of necrotic tissue and nonadherent graft. Vancomycin and cefepime were given as preoperative antibiotics (Figure 1). A biodegradable temporizing matrix (BTM) was placed with vacuum-assisted closure for negative-wound pressure therapy (NPWT). BTM and NPWT were maintained for one month until he was regrafted with autologous STSG. Preoperatively, cellulitis was observed on the right upper extremity, and oral linezolid with IV cefepime was initiated (Figure 1). For the remainder of his hospital course, his wounds were managed conservatively with regular dressing changes and topical nystatin powder, mafenide irrigation, mupirocin ointment, and bacitracin zinc-polymyxin B ointment (Figure 1). After a 73-day hospitalization in the inpatient burn unit, he was discharged to a skilled nursing facility in stable condition. Following discharge, he continued to have poor graft take on his bilateral elbows (Figures 2, 3). However, given the repeated graft failure on his bilateral elbows, a conservative non-operative approach was chosen for further outpatient management. Mupirocin and Vaseline gauze were applied daily with Tubigrip for vascular support. Given his challenges in transportation, he continues to follow up with a wound center closer to his home and, to our knowledge, is doing well.

## Discussion

*Providencia*, included alongside *Acinetobacter baumanii* and *Pseudomonas aeruginosa* in the *Enterobacteriaceae *group, is classified as Critical Priority 1 on the World Health Organization’s global priority pathogens list for research and development of new antibiotics, highlighting their international significance [14]. *Providencia *species may have a natural resistance to β-lactams due to the inducible expression of AmpC β-lactamases and, therefore, may appear sensitive to susceptibility testing despite innate resistance mechanisms. Spontaneously derepressed AmpC mutants may lead to subsequent β-lactam treatment failure [8]. Although clinical evidence is limited with *Providencia *species specifically, resistance may rarely develop following the initiation of β-lactam antibiotics, including certain third-generation cephalosporins [15]. Increasing rates of multi-drug resistant species have been identified as carbapenemase, and ESBL-producing strains have been increasingly isolated in epidemiological studies [3,5,6]. Interestingly, medical device reporting (MDR) strains of *P. rettgeri* have been described as early as the 1970s, as hospital units encountered difficulty containing outbreaks of broadly resistant strains of *P. rettgeri* [10]. The *Providencia rettgeri* strain identified in our patient’s burn wounds was resistant only to ampicillin and cefazolin on the antibiogram (Table 1). The AmpC activity of the *P. rettgeri* strain in our case report is unclear as cefoxitin and cefotetan were not included in the sensitivity results, nor were other β-lactam or β-lactamase inhibitor combination drug susceptibilities tested. There were no other *Providencia *species isolated in our burn unit aside from the patient described.

In wound infections, *Providencia *species generally occur as a polymicrobial infection, co-existing with other microbes [1,2]. In our patient’s case, *P. rettgeri* was isolated alongside MRSA and MSSA. We attribute the rapid rejection and overt necrosis of his previously adherent skin graft to the aggressive polymicrobial infection. In another case of polymicrobial infection with *P. rettgeri*, a man with tophaceous gout presented with multiple wounds from ruptured tophi with co-occurring *P. rettgeri* and *P. aeruginosa*. A synergistic mechanism has not been elucidated, but it has been postulated that colonization with other microbes may lead to attenuation of local immune responses and trigger the formation of a biofilm, producing an environment where the opportunistic *P. rettgeri* may proliferate [2]. Only one other report describes STSG following *Providencia rettgeri* infection. The patient was a 62-year-old woman with* P. rettgeri* infection secondary to snakebite, and the authors reported a successful outcome with an uncomplicated postoperative course [16].

The differential diagnosis of a chronic, nonhealing postburn wound with repeated graft failure is complex, encompassing infectious, vascular, rheumatologic, nutritional, and inflammatory etiologies [11,17]. In the absence of infection, vascular abnormalities are most common. However, alternative diagnoses, including immunologic and rheumatologic etiologies, must be considered [17]. In our patient’s case, given his continued graft rejection despite antibiotic therapy and the general appearance of his wound, there was a concern for post-surgical pyoderma gangrenosum (PG). PG is a clinical diagnosis that is supported by the presence of wounds that paradoxically worsen following debridement, that improve the following immunosuppression with steroids, and that exhibit significant neutrophilic inflammation on tissue biopsy [17]. Albeit rarely reported in post-burn injuries, post-surgical PG may be non-specific, in appearance, and consideration must be made by the burn surgeon in the event of atypical nonhealing wounds [18]. Consideration was given to PG; however, following a nonspecific biopsy result showing only mild neutrophilic inflammation, PG was considered less likely to be the underlying reason for our patient’s impaired healing than ecthyma.

Social determinants of health in a burn population

Despite the importance of support and follow-up care, burn patients face significant barriers in attending follow-up appointments, highlighting the specific impact of social determinants of health (SDOH) in this unique patient population. A retrospective study by Solomon et al. identified that over 25% of patients did not attend any follow-up appointments following a burn injury. Through a survey tool, they identified homelessness, drug use, and increased distance of travel to the clinic as the factors most associated with follow-up failure. While 60.8% of patients reported feeling confident in their ability to attend a follow-up appointment, the remaining 39.2% of patients reported obstacles that could prevent them from attending their appointment. Transportation was the most common obstacle, affecting 41.8% of patients with perceived barriers to attending an appointment [19]. A significant portion of the burn patient population has an inconsistent follow-up, as up to 56% of burn patients missed at least one follow-up appointment in a recent retrospective analysis [20]. Patients who did not attend any appointments typically had smaller burns, were more likely to be homeless, and have associated drug dependence. Distance traveled was also a significant factor, as those who missed appointments were more likely to have traveled farther, with an average living distance of 70.2 miles in those with missed appointments compared to 52.5 miles in the group without missed appointments [20]. Our patient lived 84 miles from our burn center with limited coverage of transportation costs by his insurance. Despite having access to wound care assistance at home, the patient’s lack of transportation and delayed follow-up likely contributed to his severe presentation and is a poignant reminder of the impact of SDOH on patient outcomes.

Attempts to bridge patient access to care have been made through advances in telemedicine and integration in burn medicine. Particularly in response to the COVID-19 pandemic, creative approaches to engaging patients through telemedicine have been implemented across many specialties. As technology is increasingly ubiquitous as most individuals have access to mobile phone services, remote phone applications are a promising technology for engaging patients outside of traditional in-office appointments. Leveraging telemedicine programs in burn care has been highly effective in streamlining initial patient triage, improving resource management, and promoting higher quality of care for patients [13]. While certainly many barriers to the implementation of telemedicine exist, including technical limitations, it represents a realistic opportunity to address a need in burn medicine for increased patient access to care resources.

## Conclusions

In this case report, we describe an elderly male with a burn injury who was impacted by delayed follow-up care with substantial graft loss and tissue necrosis secondary to a polymicrobial wound infection with *Providencia rettgeri*. While *P. rettgeri* has been previously described in the context of wounds, to our knowledge, this is the first description of *P. rettgeri's* complicating skin graft taken after a burn injury. With this report, we hope to increase the awareness of this infrequently encountered organism. *P. rettgeri* can have inducible AmpC expression, leading to innate resistance to β-lactams, despite appearing sensitive on susceptibility testing. Antibiotic selection is crucial and unintuitive given inducible AmpC expression, and specialized testing is recommended when *Providencia *species are isolated to ensure appropriate antibiotic selection. Antibiotic selection may be further complicated by the resistance patterns of other co-existing organisms. Additionally, our case report highlights the significance of SDOH, such as transportation in the burn patient population. As SDOHs are essential components of holistic patient-centric care, we hope to emphasize the importance of identifying potential barriers early to ensure appropriate patient follow-up. Given the significant access barriers that patients encounter, telemedicine is a promising approach to improve follow-up care in the burn patient population.
